# Editorial: Physiological response to exercise-induced stress and stressful environmental stimuli: insights from systems biology

**DOI:** 10.3389/fvets.2024.1369154

**Published:** 2024-02-16

**Authors:** Katia Cappelli, Morteza Hosseini-Ghaffari, Vincenzo Lopreiato, Samanta Mecocci

**Affiliations:** ^1^Department of Veterinary Medicine, University of Perugia, Perugia, Italy; ^2^Institute of Animal Science, University of Bonn, Bonn, Germany; ^3^Department of Veterinary Sciences, University of Messina, Messina, Italy

**Keywords:** exercise-induced stress, environmental stresses, horse, genomic response, omics, livestock, resilience

Exercise stimulates metabolic and structural adaptations that primarily affect the musculoskeletal, cardiovascular, respiratory, endocrine, and immune systems. Training modulates athletic performance and is important for reducing inflammation and preventing disease that can be caused by strenuous exercise (1). The horse is an optimal model organism often used to study the genomic response to training-induced stress because it is naturally capable of athletic performance and has a relatively homogeneous genetic and environmental background (2).

In livestock, environmental stressors (e.g., heat stress, diet, and housing) can have a negative impact on health and production by impairing the immune system, among other factors (3). Organisms employ behavioral or physiological mechanisms to cope with a disruption of homeostasis. Identifying specific resilience markers could provide an opportunity to integrate resilience into breeding objectives, which would have significant benefits over management improvement.

However, the precise cellular mechanisms underlying adaptation to exercise-induced stress and the mechanisms that allow resilient animals to respond to environmental stressors are not yet fully understood. This Research Topic aimed to collect high-quality original research that addresses the molecular mechanisms of cellular responses to stressful stimuli that may affect livestock. Five scientific articles have been published as part of this Research Topic collection.

Grzędzicka et al. investigated exercise endocrinology in horses and evaluated the differences in testosterone, cortisol, and the ratio of testosterone to cortisol (T/C; anabolic index) in response to a single training session in two types of equine sports: endurance (*n* = 12) and racing (*n* = 32). Blood samples were taken before and after exercise. Overall, T increased 2.5-fold after race training in experienced racehorses and decreased in endurance horses regardless of fitness level. In endurance horses, there was a significant decrease in T/C after training in inexperienced horses. Interestingly, inexperienced racehorses showed a decrease in T/C, whereas experienced racehorses showed an increase in T/C after training. The study supports the T/C ratio as a potentially reliable indicator of fitness status, especially in racehorses, and is used as a marker for performance and adaptation.

High-intensity interval training (HIIT) has recently gained popularity among human athletes and is defined as intermittent periods of intense exercise (>80% of maximal oxygen consumption, VO_2max_) separated by recovery periods, while sprint interval training (SIT) is characterized by efforts at an intensity that elicits >100% VO_2max_ (4). These types of interval training have been reported to induce similar or greater physiological adaptations compared to moderate-intensity continuous training (MICT) at lower durations in humans. Mukai et al. investigated whether HIIT or SIT induces greater physiological and skeletal muscle responses compared with MICT in horses. Eight trained thoroughbred horses were enrolled in the study and completed three treadmill exercise protocols, mimicking the three physical efforts. Despite the same running distance, HIIT and SIT led to exacerbating arterial hypoxemia and lactic acidosis compared with MICT. In addition, in the gluteus medius muscle, HIIT activates the AMPK signaling cascade, and HIIT and SIT increase mitochondrial biogenesis and angiogenesis, whereas MICT did not induce significant changes in these pathways. The study encourages the use of high-intensity interval training as a new training strategy for thoroughbred horses due to the greater physiological and skeletal muscle responses it elicits.

Interestingly, Ebisuda et al. considered both exercise-induced and environmental stress to assess the molecular changes. Heat acclimatization or acclimatization training in horses is practiced to reduce physiological stress and improve exercise performance in the heat, which may refine skeletal muscle metabolism. Investigating the hypothesis that exercise in hot conditions induces greater changes in heat shock proteins and mitochondrial signaling in equine skeletal muscle than training in cool conditions, 15 trained thoroughbred horses were divided for a treadmill test under cool conditions (COOL, 12.5°C; *n* = 8) or hot conditions (HOT, 29.5°C; *n* = 7). There were no significant differences between the two groups in peak heart rate and plasma lactate concentration. At the same time, HSP-70, PGC-1α, HIF-1α, and PDK4 mRNA in the mid-gluteal muscle increased significantly only in HOT 4 h after exercise. Acute exercise in a hot environment promotes the protective response to heat stress (HSP-70), mitochondrial biogenesis (PGC-1α and HIF-1α), and fatty acid oxidation (PDK4).

Concerning livestock, this Research Topic has gathered two studies on the animal welfare of pigs. D'Alessandro et al. investigated the effects of breed and diet on weight gain and levels of inflammatory and immune markers. Twenty Landrace x Large White (LxLW) and 20 Nero Siciliano (NS) pigs were allocated to one of two dietary treatments: (1) control diet and (2) control diet supplemented with liquid whey for 2 months. Consumption of liquid whey by the test group of pigs resulted in reduced levels of WBC, haptoglobin, C-reactive protein, α-, β1-, and β2-globulins and increased levels of albumin in both breeds. Overall, autochthonous breeds had higher resilience to farming conditions compared with allochthonous breeds. In addition, a certain anti-inflammatory effect of liquid whey was reported, probably due to its content of natural bioactive substances.

The effect of an alternative housing system on enhancing cognitive resilience and promoting the pigs' welfare was evaluated by Parois et al., enrolling a total of 96 piglets from two contrasted housing systems [alternative housing system (AHS) vs. conventional system (CONV)]. The AHS pigs showed lower cortisol levels and tear-staining areas before the challenge, demonstrating overall better welfare due to the alternative housing conditions. During the challenge, AHS pigs had a lower heart rate, higher heart rate variability, and higher vagal activity than the CONV pigs, which might indicate a reduced sensitivity to the stressor. AHS pigs appeared to have a better long-term memory tested in a maze. Providing social and environmental enrichment appears to be beneficial for pig welfare. Its effects on cognitive resilience still need to be proven.

In summary, the presented research articles increased the knowledge on exercise and environmental stress response, presenting new insights for the further development of a more sustainable livestock system, which is able to reduce the feed food competition and face the growing interest of new consumers. Although the collected research deals with horses concerning exercise stress, as expected, only some issues relating to pigs as farm animals have been addressed. It should be pivotal to investigate in other animal species the stressful environmental conditions that farm animal species are increasingly suffering from as a result of unstoppable global warming. Moreover, proceeding toward these kinds of studies with a systems biology approach through the multi-omics analysis coupled with cutting-edge technologies (e.g., IoT sensors) and advanced computational analyses could help to determine better the molecular mechanisms that can be used for coping strategies.

## Author contributions

KC: Writing—original draft, Writing—review & editing. MH-G: Writing—review & editing. VL: Writing—review & editing. SM: Writing—original draft, Writing—review & editing.
